# Gendermetrics of cancer research: results from a global analysis on lung cancer

**DOI:** 10.18632/oncotarget.22089

**Published:** 2017-10-26

**Authors:** Michael H.K. Bendels, Dörthe Brüggmann, Norman Schöffel, David A. Groneberg

**Affiliations:** ^1^ Division of Computational Medicine, The Institute of Occupational, Social, and Environmental Medicine, Goethe University, Frankfurt, Germany; ^2^ Department of Obstetrics and Gynecology, Keck School of Medicine of University of Southern California, Los Angeles, California, USA

**Keywords:** sex, bibliometry, academic, authorship, citation

## Abstract

**Background:**

Cancer research is critically dependent on a continuous recruitment of junior research staff that devotes its academic life not only to clinical duties but also to basic and translational research. The present study aims to elucidate the success concerning gender equality in cancer research in the last decade (from 2008 to 2016) with lung cancer as the target parameter.

**Materials and Methods:**

On the basis of the Gendermetrics Platform, a total of 19,724 articles related to lung cancer research were analyzed. The key method was the combined analysis of the proportion of female authorships and the female-to-male odds ratio for first, co- and last authorships. The distribution of prestigious authorships was measured by the Prestige Index.

**Results:**

31.3% of all authorships and 35.2% of the first, 32.2% of the co- and 22.1% of the last authorships were held by women. The corresponding female-to-male odds ratio is 1.22 (CI: 1.18–1.27) for first, 1.19 (CI: 1.16–1.23) for co- and 0.59 (CI: 0.57–0.61) for last authorships. Women are underrepresented at prestigious authorships compared to men (Prestige Index = −0.22). The female underrepresentation accentuates in articles with many authors that attract the highest citation rates.

**Conclusions:**

While the current system promotes early career promotion of women, men still outnumber women in leadership positions. However, this male-female career dichotomy has been narrowed in the last decade and will likely be further reduced in the next decade.

## INTRODUCTION

Lung cancer research is currently characterized by a tremendous amount of new insights and approaches ranging from genetics [1] over novel treatment options [2] to diagnostics [3–5] and prevention [6, 7]. In the past two decades, there has been an increased focus on gender differences in health and disease [8]. Currently, lung cancer is known to be the most common cause of cancer death in US-American women. It accounts for more than one quarter of all cancer deaths [9]. Historically, this cancer has been viewed as a male disease, but during the past half century, a dramatic increase in the incidence of lung cancer in women has been reported [9]. With gender differences become more and more important concerning pathogenesis for lung cancer [10, 11], also the questions evolves of the role of women in lung cancer medicine and research [12]. Research activities for cancer issues have recently been assessed by a number of studies [13–15]. For lung cancer Aggarwal et al. analyzed a total of 32,161 lung cancer research articles from 2085 different journals [16]. They found out that lung cancer research represented only 5.6% of overall cancer research in 2013, a 1.2% increase since 2004 [16]. They also reported that the commitment to lung cancer research has fallen in most countries apart from China and shows no correlation with lung cancer burden [16]. A review of key research types demonstrated that diagnostics, screening, and quality of life research represent 4.3%, 1.8%, and 0.3% of total lung cancer research, respectively [16]. The leading research types were genetics (20%), systemic therapies (17%), and prognostic biomarkers (16%) [16].

With regard to this bibliometric study, we here assess the integration of women in the field of lung cancer research by analyzing their representation in scientific authorships. Conceptually, we exploit the fact that the prestige of authorships follows, by convention, a ranked order with a higher reputation of first and last authorships and a lower reputation of co-authorships [17, 18]. Moreover, authorships also reflect the hierarchical structures of the underlying research community, as early-career researches usually publish as first or co-authors and senior researches preferably as last authors [17, 19].

Methodically, we used the Gendermetrics Platform [20] to analyze the representation of 121,407 male and female authorships from 19,724 English original articles related to lung cancer, published between January 1, 2008 and September 20, 2016. By considering the different prestige of first, co- and last authorships, we draw conclusions about the distribution of prestigious authorships between the two genders, as previously shown in Bendels et al. [18, 21]. The analysis evaluates global status, temporal development and future perspectives, differences across continents and countries, scholarly productivity, citation rates and finally, the role women tend to have in articles with many authors.

## RESULTS

### Female authorships on the global level

**Figure 1:**
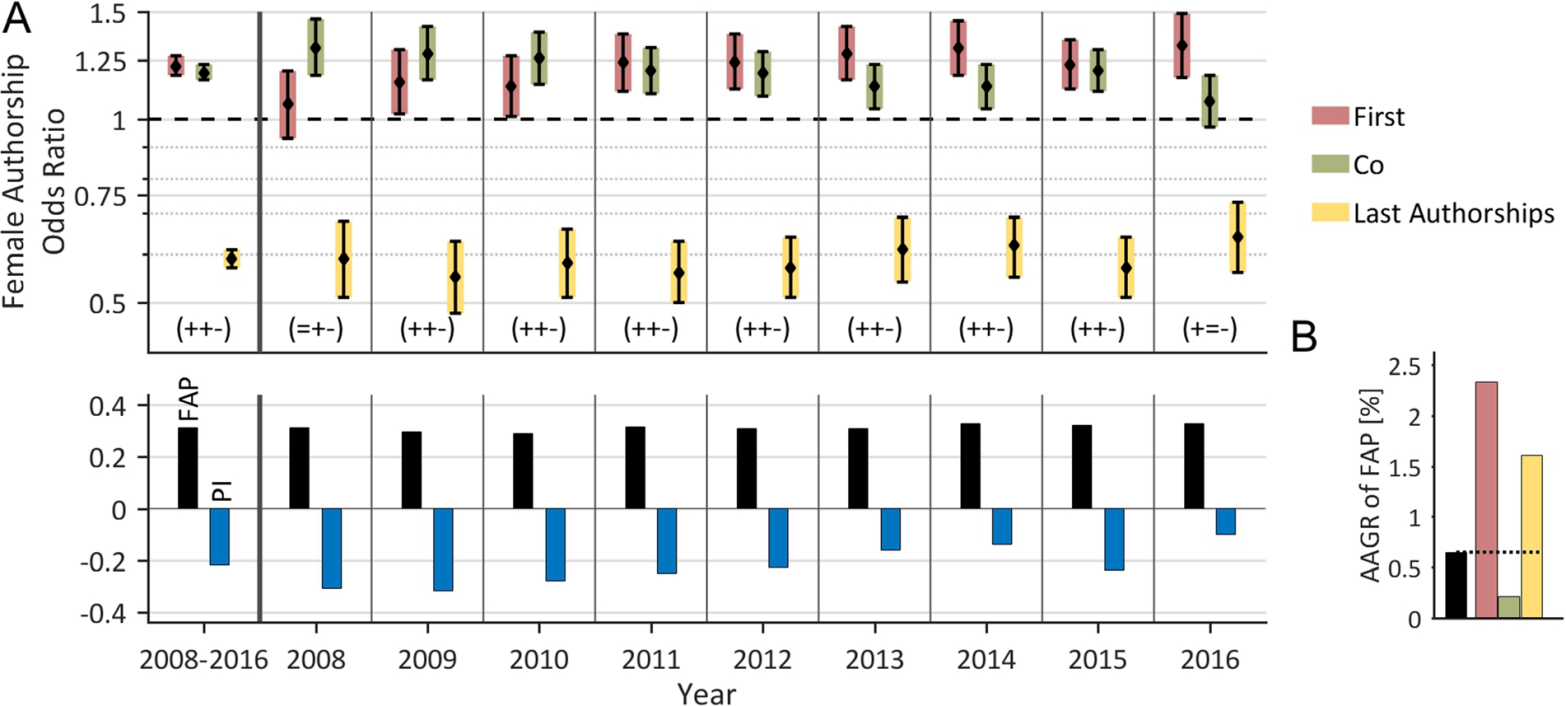
Time trend of female authorships on the global level (**A**) The proportion of female authorships (FAP, bottom), the pattern of female authorship odds (FAOR with FAOR-tuple, top) and the associated Prestige Index (PI) are depicted by year and averaged over time. The very time-stable and unbalanced FAOR-patterns are predominantly characterized by significant (*P* < .05) higher female odds to be first- or co-author and lower odds of assigning a last authorship compared to men (FAOR-tuple (+, +, −)). The FAR is on average 31.3%. The Prestige Index is negative pointing to a lack of prestigious authorships hold by women. (**B**) The FAP shows only a minor increase during the period as indicated by its annual growth rate (AAGR) of 0.65%. The highest AAGR was revealed for the first author position (2.33%).

In a first step, we analyzed the temporal evolvement of female authorships in the field of lung cancer on a global level (Figure 1). We determined an underrepresentation of female authorships with a FAP of 31.3%. The sub-classification of female authorships shows relatively more female first (35.2%) and co-authorships (32.2%) and a substantially less fraction of last authorships (22.1%). The FAP grows from 31.4% in 2008 to 32.8% in 2016; the AAGR is 0.65% (Figure 1B). The highest AAGR was revealed for first authorships (2.33%), followed by last authorships (1.61%) and co-authorships (0.21%).

The global pattern of FAORs is characterized by the FAOR-tuple (+, +, −), i.e. the female-to-male odds ratios (FAORs) are characterized by significantly higher female odds to secure first (1.22, CI: 1.18–1.27) and co-authorships (1.19, CI: 1.16–1.23) and by significantly lower female odds to hold last authorships (0.59, CI: 0.57–0.61). Men have an approximately 1.7 higher odds to secure last authorships compared to women. The identified FAOR-pattern is almost constantly present over the whole evaluation period. The imbalance in authorships odds between both genders is reflected by the Prestige Index, which is on average −0.22, thus indicating negative female odds to secure prestigious authorships compared to men. The Prestige Index shows an increase from −0.31 in 2008 to -0.10 in 2016 reflecting a substantially improvement of female odds to secure prestigious authorships (Figure 1A, bottom).

### Differences across continents

On the level of continents the FAP ranges from 15.0% in Asia to 45.2% in South America (Table 1). The FAOR pattern ranges from unfavorable in Asia with the tuple (=, +, −) to more favorable in Australia & Oceania with the tuple (+, =, −). In Australia & Oceania a negative FAOR for last authorships is numerically compensated by a positive FAOR for first authorships resulting in a gender-neutral distribution of prestigious authorships (Prestige Index = 0). In all other continents, the Prestige Index is negative ranging from -015 in North America to −0.70 in Asia.

**Table 1:** Classification of continents

Continent Name	FAP	FAOR Triplet	Prestige Index	#Articles	#Authorships
Australia & Oceania	40.0%	(+, =, −)	0	426	1524
North America	33.8%	(+, +, −)	−0.15	7891	40231
South America	45.2%	(=, =, −)	−0.20	300	1145
Europe	38.2%	(+, +, −)	−0.39	8421	39533
Asia	15.0%	(=, +, −)	−0.70	3616	25192
Africa	−	−	−	63	100
Central America	−	−	−	9	10

The FAP/FAOR-classification was only conducted for continents with at least 750 male and female authorships. Continents were descendingly ordered by the Prestige Index.

### Differences across countries

When we refined our analysis from continental- to country-specific level we identified a wide range of FAPs in lung cancer research ranging from 12.4% in Japan, over 33.4% in the U.S. to 47.7% in Spain (Table 2). The unfavorable FAOR-triplet (=, +, −) was found in Japan, Spain, Germany, Turkey, Italy, Greece, and France, whereas the countries Sweden and Canada are characterized by the favorable FAOR-triplet (+, =, =). Remarkably, in almost all countries - with the exception of Sweden and Canada - women have lower odds to be a last author compared to their male counterparts.

**Table 2:** Classification of countries (descendingly ordered by the Prestige Index)

Country Name	FAP	FAOR Triplet	Prestige Index	#Articles	#Authorships
Sweden	39.8%	(+, =, =)	0.3	258	899
Netherlands	31.0%	(+, −, −)	0.22	714	3384
Canada	39.0%	(+, =, =)	0.11	903	3444
Australia	40.0%	(+, =, −)	0	426	1524
Denmark	42.2%	(+, =, −)	-0.08	272	1072
United States	33.4%	(+, +, −)	-0.18	6988	36787
Poland	45.3%	(+, =, −)	-0.24	329	1347
United Kingdom	38.3%	(+, +, −)	-0.31	1035	3961
Switzerland	31.5%	(+, =, −)	-0.32	288	950
France	39.3%	(=, +, −)	-0.42	817	4731
Belgium	31.2%	(=, =, −)	-0.57	282	950
Austria	28.7%	(=, =, −)	-0.58	185	795
Greece	37.0%	(=, +, −)	-0.63	231	1060
Italy	44.2%	(=, +, −)	-0.7	1095	7380
Turkey	36.0%	(=, +, −)	-0.71	329	1718
Germany	26.5%	(=, +, −)	-0.74	1167	6210
Spain	47.7%	(=, +, −)	-0.92	637	3196
Japan	12.4%	(=, +, −)	-1.14	2981	22477

Interestingly, no top 15 country can provide gender-neutrality regarding authorship odds. The highest *Prestige Indices* were found in Sweden (0.30), the Netherlands (0.22), and Canada (0.11). Australia provides gender-neutrality regarding the distribution of prestigious authorships between the two genders (Prestige Index = 0). Lowest *Prestige Indices* were determined for Germany (−0.74), Spain (−0.92), and Japan (−1.14). We reveal no significant correlation between the FAP of a country and its Prestige Index (R(16)=0.35, *P* > .05).

### Female authorships by authors per article

**Figure 2:**
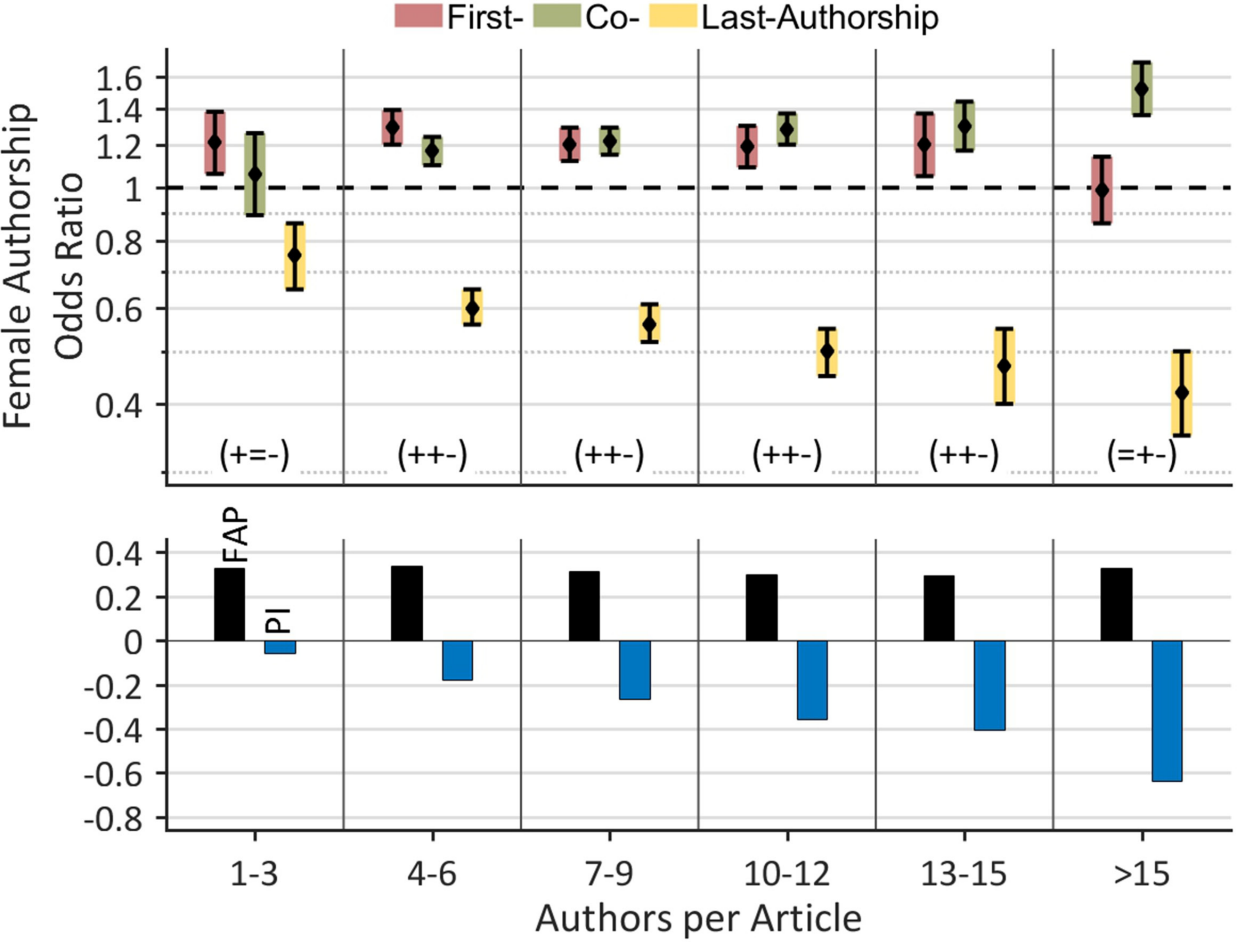
Female authorships by authors per article The more authors contribute to an article, the the lower is the representation of women at prestigious authorships, whereas the FAP remains almost constant.

We also applied the FAP/FAOR-classification to investigate the role women tend to have in articles with many authors, e.g. collaboration articles (Figure 2). The FAP does not show any significant changes related to the number of authors per article. In particular, the FAP remains at a constant level of 32.5% for articles with 1–3 and for articles with more than 15 authors. By contrast, the FAOR for prestigious first or last authorships decreases (first: 1.21 to 0.99, last: 0.75 to 0.42), whereas the FAOR for less prestigious co-authorships increases (1.06 to 1.53); the differences for co- and last-authorships were statistically significant. Overall, this leads to a continuous decrease of the Prestige Index from −0.06 for articles with 1–3 authors, to −0.64 for articles with more than 15 authors. To conclude, the more authors contribute to an article the statistically lower is the representation of women at prestigious authorships.

### Citation and productivity analysis

**Figure 3:**
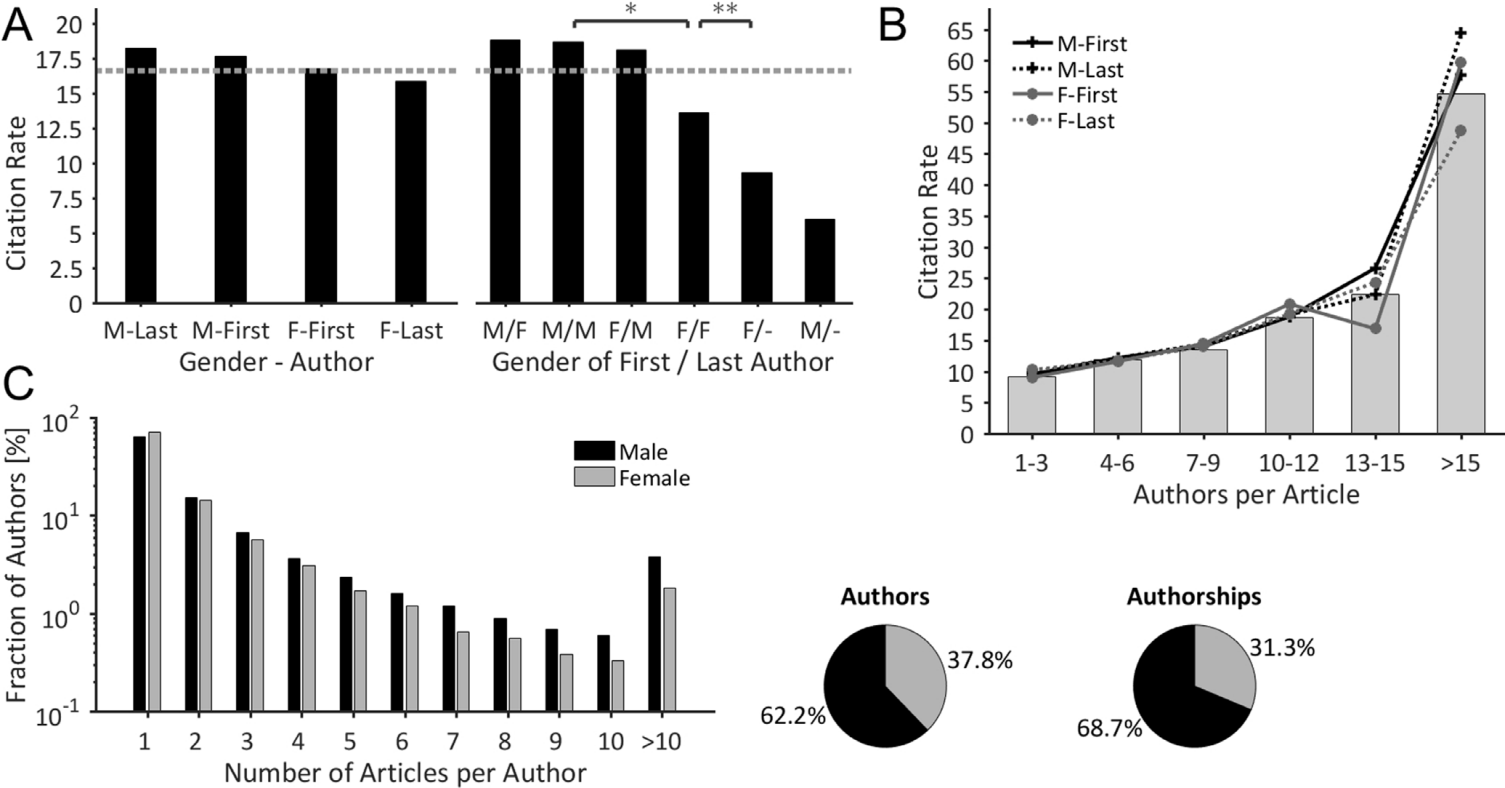
Gender-specificity of citations & scholarly productivity (**A**) The descendingly ordered citation rates document that male-authored articles are more frequently cited than female-authored articles. The dotted line characterizes the mean citation rate of 16.6 citations/article (Kruskal-Wallis test, (^*^):*P* < .05 (^**^):*P* < .01). (**B**) Average citation rates of both, ungrouped articles (bars) and articles that were grouped by the gender of their key authorships (lines), plotted with respect to the number of authors. Statistically, the citation rate of an article is higher the more authors are involved. No significant gender-specific differences were present in citation rates up to an author count of 12 authors per article. (**C**) Gender-specific distribution of the number of articles per author. Women dominate the sub-group ‘author has 1 article’. All other sub-groups show a clear over-representation of male authors, which accentuates with increasing productivity levels. Overall, male authors have a higher productivity, as 62.2% male authors are responsible for 68.7% of all authorships.

In a last step, the citation rates of lung cancer articles were analyzed with respect to the gender of the first and last author (Figure 3). The analysis reveals that lung cancer articles with a male author at the first or last position are on average more frequently cited than articles with female authors. The citation rates range from 15.9 citations for articles with a female last author to 18.2 citations for articles with a male last author. However, the differences are not statistically significant (Kruskal-Wallis test, *p* >.05). The mean citation rate is 16.6 citations per article. The analysis of combined (first/last) authorships reveals that articles with at least one male first or last author are cited above-average, whereas articles with two female key authors or single author articles - regardless of the authors’ gender - are cited below average (Figure 3A, right). Interestingly, single author articles published by women are more frequently cited than those written by men (9.3 vs. 6.0 citations per article).

Statistically, the citation rate of an article increases the more authors are involved (Figure 3B), as e.g. the average citation rate of articles with 1–3 authors is 9.1, whereas articles with more than 15 authors are cited on average 54.7 times. No significant gender-specific differences were present in citation rates up to an author count of 12 authors per article. By contrast, differences exist for articles we more than 15 authors, particularly between articles with male and female last authors that are cited on average 64.4 and 48.7 times, respectively.

The analysis reveals marked differences in scientific productivity between the two genders: Women clearly dominate the sub-group with the lowest productivity (‘author has one single article’), as 70.3% of the female authors, but only 63.4% of the male authors had published a single article in our dataset (Figure 3C). By contrast, for all other sub-groups - with authors that published more than one article – we found a clear over-representation of male authors, which accentuates with increasing productivity. In particular, the sub-group of most productive authors is clearly dominated by men, as 3.8% of the male authors but only 1.8% of the female authors published more than 10 articles [18]. In total, 62.2% male authors secure 68.7% of all authorships in our data set, thus indicating a higher productivity of the male scholars.

## DISCUSSION

### Male-female career dichotomy

In this descriptive study, we applied a bibliometric approach to investigate the representation of women in lung cancer research. The global FAP of 31.3% corresponds approximately to those estimated for the whole area of science by Lariviere et al. in 2012 (30%) [22]. By contrast, the value is significantly lower than the FAPs revealed for six high-impact medical journals (34.0%) [23] and the research fields of dermatology (43.0%, unpublished data), epilepsy (39.4%) [18], schizophrenia (37.6%) [21], and stroke medicine (36.3%, unpublished data) for the same period.

Women are uneven distributed across the different authorships: We found a relative overrepresentation of women at first and co-authorships and a female underrepresentation at last authorships compared to men (FAOR-pattern (+, +, −)). Evidently, this pattern reflects the well-known male-female dichotomy in scientific careers with many female early-career researchers at lower levels in the hierarchy and just a few women at leadership positions [18, 21, 24–28].

Moreover, the FAOR-distribution reveals that women are underrepresented at prestigious first and last authorships compared to men. As is the case for other research areas [18, 21], the high FAOR for first-authorships does not compensate the unfavorable FAORs for co- and last authorships [18]. This is a very important result, since academic publishing at prestigious authorships is the key element of career advancement in science [21, 29–31]. Reasons for the relative overrepresentation of female co-authorships - extensively discussed by West et al. [32] - range from high influx of female early-career researchers in recent decades, over an unsuccessful female negotiating for more prestigious authorships, to speculations regarding a lower contribution of women to an article [18].

### Position affects productivity and citation rate

As is the case in many other disciplines [18, 21, 22, 24, 26, 27, 33], women publish fewer articles than men, as 37.8% female authors are responsible for 31.3% of the authorships in lung cancer research [18]. This mismatch lies in a comparable range with other medical disciplines like epilepsy research where 43.8% female authors hold 39.4% of the authorships [18] or schizophrenia research where 45.5% female authors are responsible for 37.6% of the authorships [21]. Regarding productivity of single authors, we were able to reproduce the marked overrepresentation of male authors at higher productivity levels, as previously shown for the field of evolutionary biology and ecology [23], epilepsy [18] and schizophrenia research [21]. One reason for the higher productivity of male authors is surely the higher output of the primarily male senior scientists [21, 28] that are often embedded into a more or less fruitful scientific network. Due to these *structural reasons* [29], the female underrepresentation at prestigious authorships accentuates in articles with many authors (Figure 2), e.g. in highly competitive collaborative articles, which usually attract the highest citations rates (Figure 3B) [34]. It is plausible to assume that this competitive displacement causes the slightly higher citation rates of articles with male key authors compared to those with female key authors, especially since articles with up to 12 authors do not exhibit any differences in citation rates between the two genders (Figure 3B). It must be emphasized that the gender-specific differences in citation rates are relatively small in comparison to other scientific fields [22, 32, 35, 36]. Methodically, the results are biased towards the early period of investigation (2008–2010) due to the time-delayed occurrence of citations (“Cited Half-Life”) [37].

### Regional aspects

**Figure 4:**
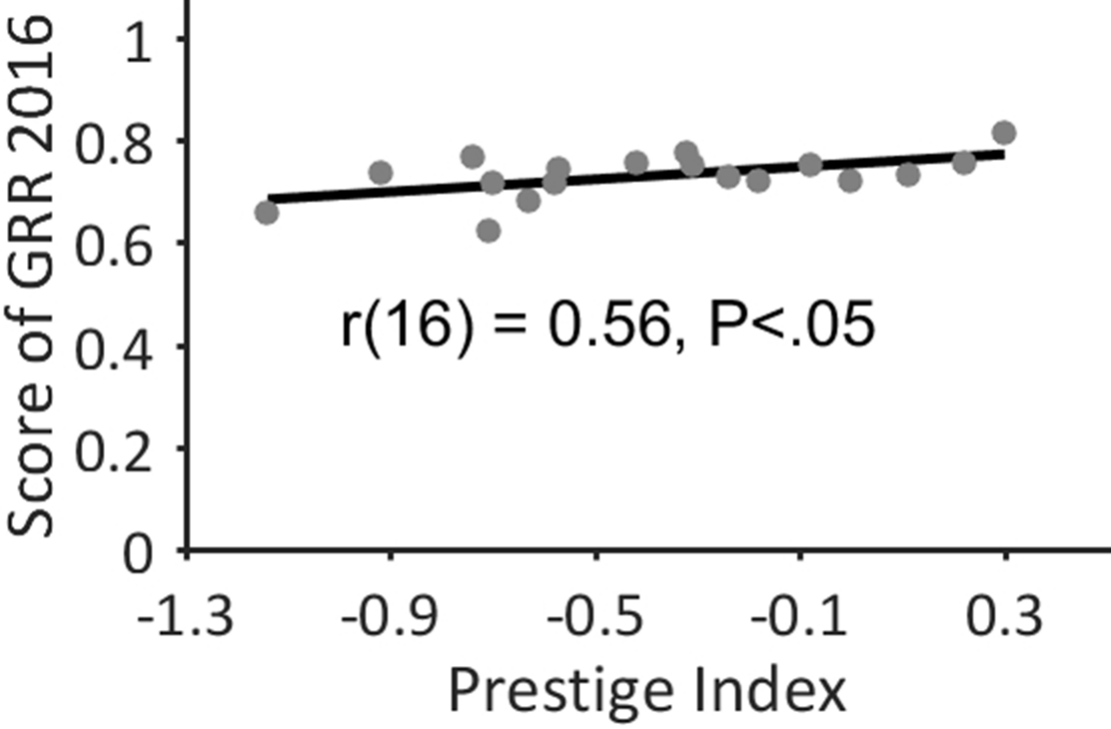
Prestige index vs. score of global gender report 2016 We reveal a large linear correlation between the Prestige Index of a country and its *Score* defined by the Global Gender Report 2016. Evidently, major regional differences stem primarily from the socio-cultural surroundings of a country and are not the outcome of discipline-specific characteristics.

We revealed significant differences in both proportion and odds ratios of female authorships among continents and individual countries. When taking the odds of securing prestigious authorships as an indicator for career advancement in science [18], Sweden, the Netherlands, and Canada provide best conditions for women. By contrast, Turkey, Germany, Spain and Japan offer optimal conditions for men in lung cancer research. These findings correlate quite well with the results of the Global Gender Gap Report 2016 [38], as we reveal a large linear correlation between the Prestige Index of a country and its *Score* defined by the Global Gender Report (r(16) = 0.56, *P* < .05; Figure 4). This suggests that the major regional differences are mainly based on socio-cultural and socio-economic conditions and are not the outcome of discipline-specific characteristics. Notably, we do not reveal a significant correlation between the FAP of a country and its Prestige Index. This finding is consistent with results from the research fields of epilepsy [18] and schizophrenia [21]. This means, countries with a high FAP may also provide disadvantageous career opportunities for women and vice versa [18]. A good example of this is Spain, where a high FAP of 47.7% is combined with the second lowest Prestige Index (-0.92) of all considered countries. The lack of correlation between FAP and Prestige Index is in contrast to the socio-dynamic theory of critical mass [39] stating that ‘with an increase in relative numbers, minority members are potentially allies, can form coalitions, and can affect the culture of the group’ [40].

Interestingly, the non-advanced integration of Japanese women (lowest FAP with 12.4%, unfavorable FAOR-pattern (=, +, −) and lowest Prestige Index of -1.14) is concomitant by a statement of the Japanese government conceding that its world standing in science and technology is falling [41]. As a consequence of this, a range of policies was recently introduced by the Japanese government to recruit top international researchers [42].

### Methodical limitations

Conceptually, we extend frequency-based approaches [43–46] by including the odds ratios of female authorships [23] as well as the different prestige of first, co- and last authorships. The fully automated bibliometric approaches ensures a fast and reliable analysis with a minimized inter-individual variability. However, as already mentioned in Bendels et al. [18], the scope of this method is limited by the absence of information regarding the scholar's academic position (e.g. Associate Professor vs. Full Professor), its academic degree, age and employment status. This information can only be assessed by questionnaires or the inspection of e.g. online profiles, as exemplified by other studies [27, 28, 45]. Another drawback of the bibliometric approach is that we had to exclude the Asia countries China, South Korea and Taiwan from the *country-specific* analysis due to the high proportion of unisex names.

## MATERIALS AND METHODS

### Data acquisition and integration

English-language research articles were acquired on 22.09.2016 from the Web of Science Core Collection by performing a title search including the terms ‘Lung Cancer’, ‘Lung Neoplasms’, ‘Lung Carcinoma’, ‘Pulmonary Cancer’, ‘Pulmonary Neoplasms’ or ‘Pulmonary Carcinoma’. The synonyms for ‘Lung Cancer’ were determined by the MeSH library (Medical Subject Headings) of the National Library of Medicine. The aim was to create a representative subset of lung cancer related articles. The study period covers January 1, 2008 to September 20, 2016, yielding 19,724 articles. The data analysis was conducted using Gendermetrics.NET [20], a SQL-Server based platform for analyzing bibliometric data with a special emphasis on gender aspects. Authors were unified by names and first names. In total, 71,129 authors from 97 countries were identified.

### Gender determination

The algorithmic author gender determination uses a data table that reliably defines the gender of 77,818 first names including unisex names, as previously described in Bendels et al. [18, 20]. Importantly, the detection algorithm generates no bias towards a higher detection ratio of male or female names in our data set. The gender detection is also numerically stable, as illustrated by Supplementary Figure 6. Journals with a detection rate below 50% male or female authors were excluded from the analysis (in total 642 journals and 8128 articles). Low journal-specific detection rates are mainly due to the predominant usage of initials preventing the correct gender determination. The proportion of detected male and female authors exhibits a small inter-annual variability, as illustrated by Supplementary Figure 2. In total, 32,105 (= 45.1%) male authors, 19,519 (= 27.4%) female authors, 9,673 (=13.6%) unisex authors and 9,832 (= 13.8%) undefined authors were determined. Unisex and undefined authors and their authorships (*N* = 41,577) were ignored in further analysis. In total, *N* = 121,407 male and female authorships form the database for the analysis. The research output of a country was determined on the basis of the associated institutions and their authorships [18]. A single author is thus able to contribute to the research output of different countries (Supplementary Figure 8). The quality of gender detection depends considerably on the authors’ country as illustrated by Supplementary Figure 3B. In order to ensure the validity of the country-specific analysis a gender detection threshold criterion for the inclusion of a country was applied (Supplementary Figure 3A). Specifically, countries with a detection fraction below 73.8% male and female authorships were excluded from this subanalysis. Among the top 20 most productive countries, the Asian countries China, South Korea and Taiwan (with a high rate of unisex names) were excluded. Please note that the threshold criterion was exclusively applied for the country-specific analysis. Supplementary Figure 1 gives a general overview of the bibliometric data. Supplementary Figure 7 summarizes the methodical steps.

### Proportion of female authorships (FAP) & female authorship odds ratio (FAOR)

In this study, three types of authorships were considered: First, co- and last authorships, whereby the term co-authorships encompasses all authorships between *one* first- and *one* last-authorship [18]. Equally distributed first and last authorships were not considered due to a lack of information. The proportion of female authorships (FAP) is defined as the quotient between the female authorship count and the total sum of male and female authorships [18]. Additionally, the authorship-specific odds ratios for female authors compared to male authors are determined (female authorship odds ratio, FAOR) with the corresponding confidence intervals at a confidence level of 95% [18]. The FAOR for first authorships is determined by considering all articles, whereas the FAORs for last and co-authorships are calculated by considering all articles with at least two or three, respectively, authorships. For systematization, a triplet was introduced in order to indicate the sign of the *significant* female odds ratio excess to secure a first, co- and last authorship. For example, the FAOR-triplet (=, −, +) indicates that women have equal odds for first and *significantly* lower and higher odds for co- and last authorships, respectively. To summarize, the FAP measures the quantitative representation of female authorships, while the three FAORs quantify the relative distribution of female authorships among the different authorships [18]. In order to achieve an adequate statistical precision in terms of small confidence intervals, the FAP/FAOR-classification is only conducted for subjects (e.g. countries) with a minimum of 750 male or female authorships.

### Prestige index

The Prestige Index measures the female odds excess of securing prestigious authorships compared to men. It is defined as the prestige-weighted average of the FAOR excess ε_t_ that is calculated over all authorship types t (i.e. for first, co- and last authorships), ε_t_ = w_t_ (FAOR_t_ – 1), if FAOR_t_ ³ 1, otherwise ε_t_ = w_t_ (1 − 1/FAOR_t_) with the weighting factor w_t_ [18]. In accordance with the higher reputation of first and last authorships compared to co-authorships, the former were graded positively (w_first_ = w_last_ = 1), whereas co-authorships are graded negatively (w_co_ = –1). In this weighting scheme, lower odds for a middle authorship increase the Prestige Index, whereas lower odds for a first or last authorship decrease the Prestige Index. A Prestige Index of 0 indicates a gender-neutral distribution of prestigious authorships, whereas a value above (below) 0 characterizes an excess (lack) of prestigious authorships held by women [18]. An alphabetic ordering of the author list was excluded by an additional test (Supplementary Figure 5).

### Analysis of data

Average annual growth rates (AAGR) were applied to measure the annual growth. The AAGRs of the article count and the FAPs were used to make a linear forecast of the temporal development of FAP, FAOR and Prestige Index for the coming decade. The linear association between FAP, Prestige Index and *Score* of the Global Gender Report [38] was evaluated by means of the Pearson correlation. The null hypothesis, whether the not normally distributed citation rates of the different article groups (Supplementary Figure 4) are drawn from the same distribution was tested by a Kruskal-Wallis and a follow-up multiple comparison test [18].

## CONCLUSIONS AND OUTLOOK

**Figure 5:**
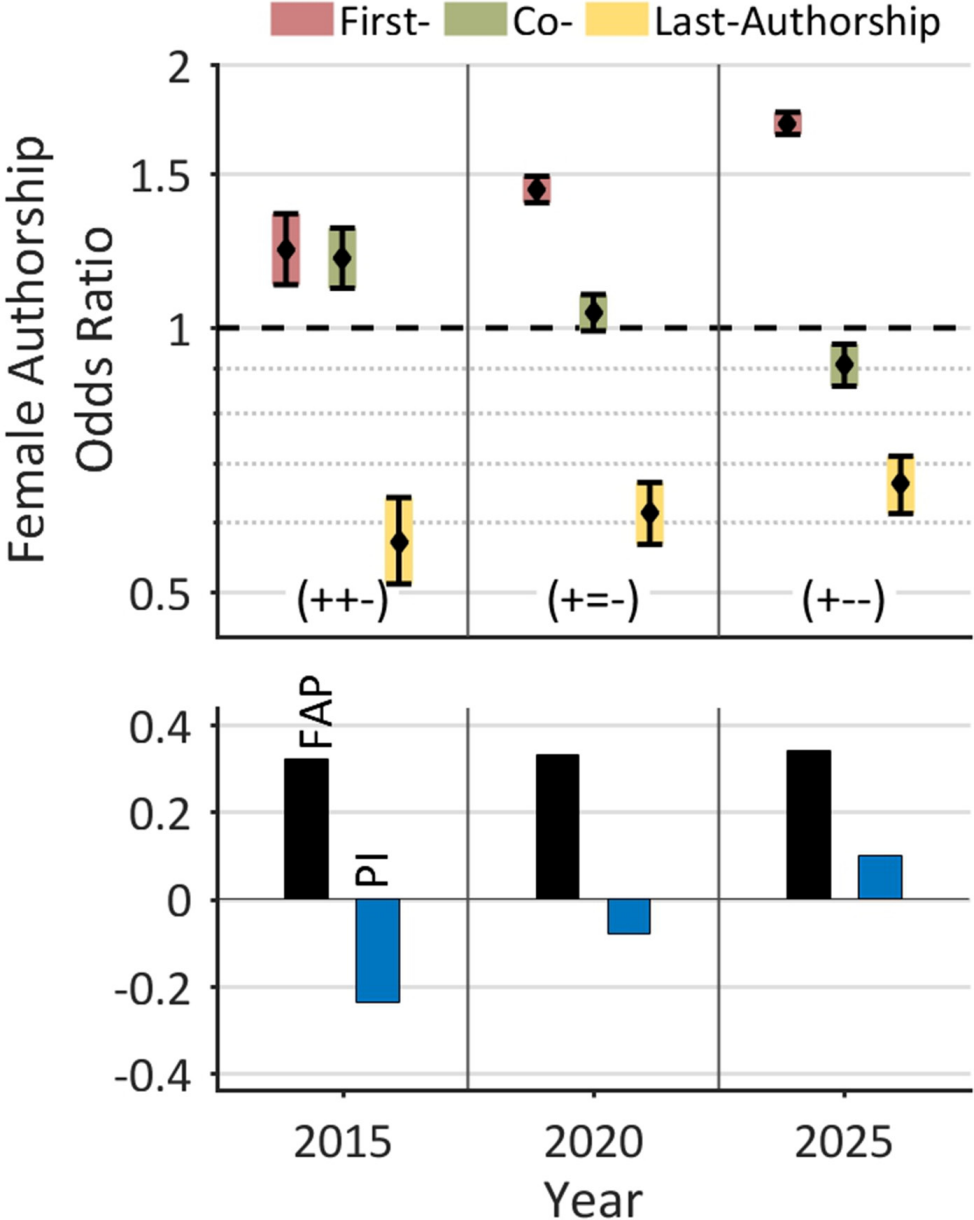
Linear projection of the development of female authorships on the global level The prognosis for the next decades forecasts only a minor increase of the FAP, but a significant improvement of female authorship odds. According to this projection, the FAOR-pattern will change from (+, +, −) to (+, −, −) and the Prestige Index will become positive.

Overall, we found a relatively low FAP compared to other medical disciplines [18, 21], a remarkable male-female career dichotomy, and an accentuated female underrepresentation at key authorships in articles with many authors. On the other hand, the analysis revealed relatively high AAGRs of the FAPs for first (2.33%) and last authorships (1.61%), a finding that reflects the catching-up process of women during the last decade. Based on this data, a quantitative prognosis of the temporal development of female authorships up to the year 2025 (Figure 5) forecasts only a minor increase of the FAP (from 32.1% 2015 to 34.3% in 2025), but a significant improvement of female authorship odds. Specifically, the prognosis forecasts an increase of the FAOR for first (from 1.23 to 1.71) and last authorships (0.57 to 0.66) and a decrease of the FAOR for co-authorships (from 1.20 to 0.91). According to this projection, the FAOR-pattern will change from (+, +, −) to (+, −, −) and the Prestige Index will become positive (from -0.24 in 2015 to 0.10 in 2025). On the basis of this forecast, we do expect a deeper integration of women in the field of lung cancer research with an increasing number of female leaderships in the next decade.

## SUPPLEMENTARY MATERIALS FIGURES AND TABLES



## Abbreviations

AAGR: Average Annual Growth Rate
FAOR: Female Authorship Odds Ratio
FAP: Proportion of Female Authorships
PI: Prestige Index
