# Supplementary material for: Gendermetrics of cancer research: results from a global analysis on lung cancer
Source: Oncotarget. 2017 Oct 26;8(60):101911–21. doi: 10.18632/oncotarget.22089 (PMC5731923; doi:10.18632/oncotarget.22089)
Supplement: Supplementary file 1 [file oncotarget-08-101911-s001.pdf]

Gendermetrics of cancer research: results from a global analysis on lung cancer

SUPPLEMENTARY MATERIALS

SUPPLEMENTARY REFERENCES

1. Bendels MHK, Brüggmann D, Schöffel N, Groneberg DA. Gendermetrics.NET: a novel software for analyzing the gender representation in scientific authoring. J Occup Med Toxicol. 2016; 11:43

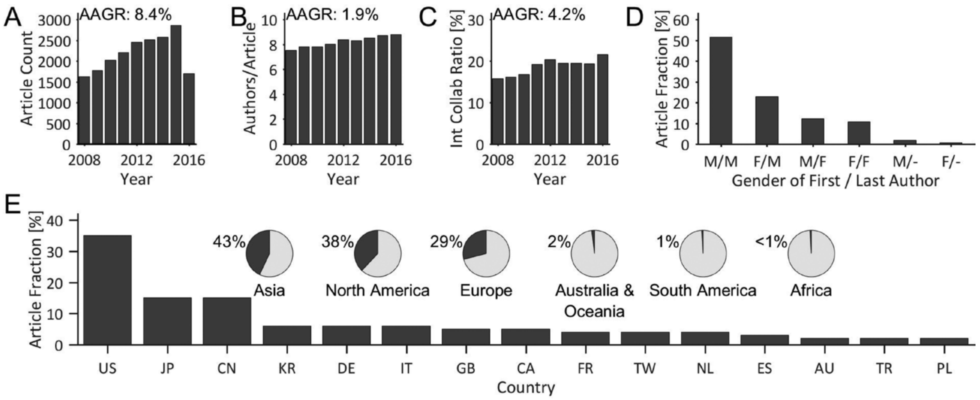

Supplementary Figure 1: Bibliometric Overview. The study period covers January 1, 2008 to September 20, 2016, yielding 19,724 articles. (**A**) The increasing relevance attached to the issue of lung cancer is reflected by the corresponding article count and its high average annual growth rate (AAGR) of 8.4%. (**B**) The number of authors per article (author-rate) increases from 7.5 authors/article in 2008 to 8.8 authors/article in 2016, which results in an AAGR of 1.9%. (**C**) The ratio of international collaboration articles increases from 15.7% in 2008 to 21.6% in 2016 with an AAGR of 4.2%. (**D**) The grouping of articles by the gender of their first and last author reveals a quantitative dominance of combined male first and last authorships (M/-, F/-: single authorships). (**E**) The fraction of articles by country (bar plot) and continent (pie chart) documents that the United States (35.0%), Japan (15.0%), and China (15.0%) and on the level of continents, Asia (43%), North-America (38%) and Europe (29%) are the most productive countries and continents, respectively. Please note that the sum of ratios is greater than one in both group classes due to international collaborations. AU = Australia, CA = Canada, CN = China, DE = Germany, ES = Spain, FR = France, GB = United Kingdom, IT = Italy, JP = Japan, KR = South Korea, NL = Netherlands, PL = Poland, TR = Turkey, TW = Taiwan, US = United States.

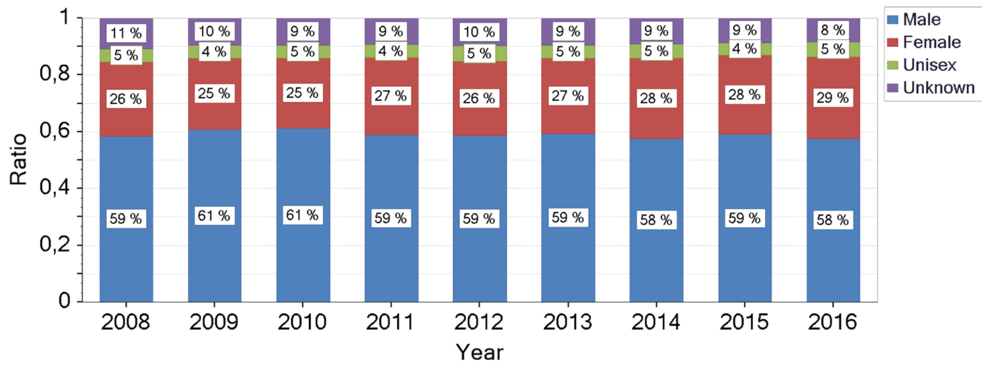

Supplementary Figure 2: Gender detection output by time. The ratios of detected male, female, unisex and undefined authorships ordered by publication year are characterized by a relatively little inter-annual variability.

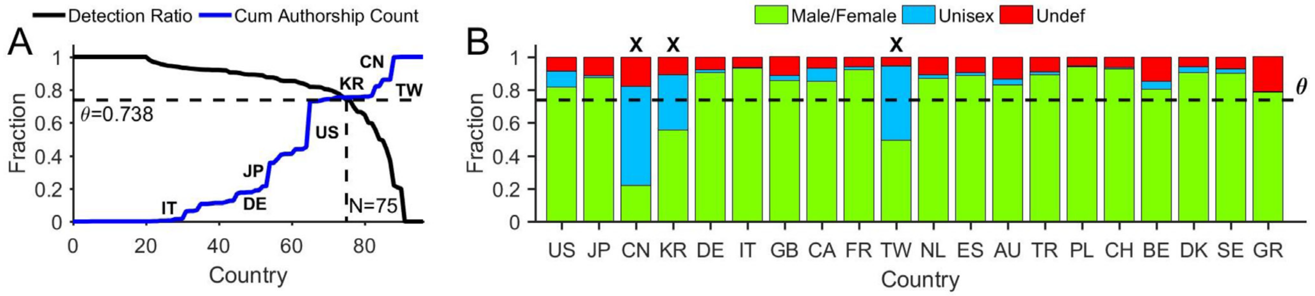

Supplementary Figure 3: Quality of Algorithmic Gender Detection by Country. (**A**) An adaptive threshold country criterion θ for the inclusion of a country in the country-specific gender analysis was defined by a ROC-like curve incorporating both detection ratio and cumulative author count , see Bendels et al. [1]. In this study, countries with a detection rate of at least $\theta = 0.738$ male + female authors (representing 73.8% of all authorships) from N=75 countries were included in the country-specific analysis. Countries with a large amount of authors are indicated by country code. (**B**) The result of the algorithmic gender detection - classified as male/female, unisex or undefined - grouped by countries that are ordered in descending order by their publication count, documents a relative high frequency of male/female authors for most of the top 20 countries, with the exception of the Asian countries China, South Korea and Taiwan. The latter countries are characterized by a high frequency of unisex names were excluded (X) from analysis due to the threshold criterion θ (dashed line). AU = Australia, BE = Belgium, CA = Canada, CH = Switzerland, CN = China, DE = Germany, DK = Denmark, ES = Spain, FR = France, GB = United Kingdom, GR = Greek, IT = Italy, JP = Japan, KR = South Korea, NL = Netherlands, PL = Poland, SE = Sweden, TR = Turkey, TW = Taiwan, US = United States.

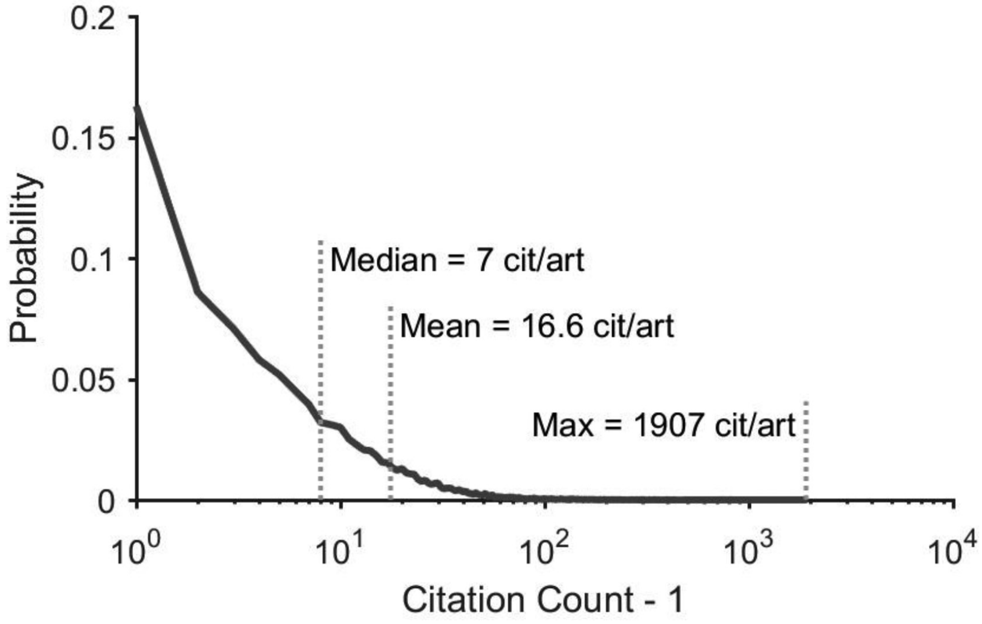

Supplementary Figure 4: Probability density function of the citation rate. The semi-logarithmic plot of the citation count per article (= citation rate) exhibits an exponential-like decreasing probability density function with a mean citation rate of 16.6 citations/article.

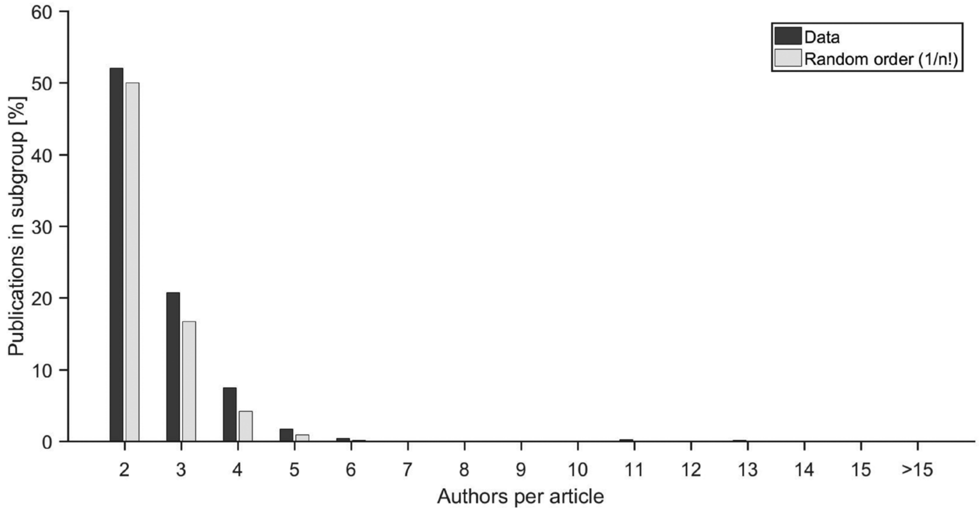

Supplementary Figure 5: Test for alphabetical ordering of the author list. The proportion of publications with an alphabetic ordered author list is depicted with respect to the authors per article (black). The values correspond very closely to those obtained for randomly ordered author lists (gray).

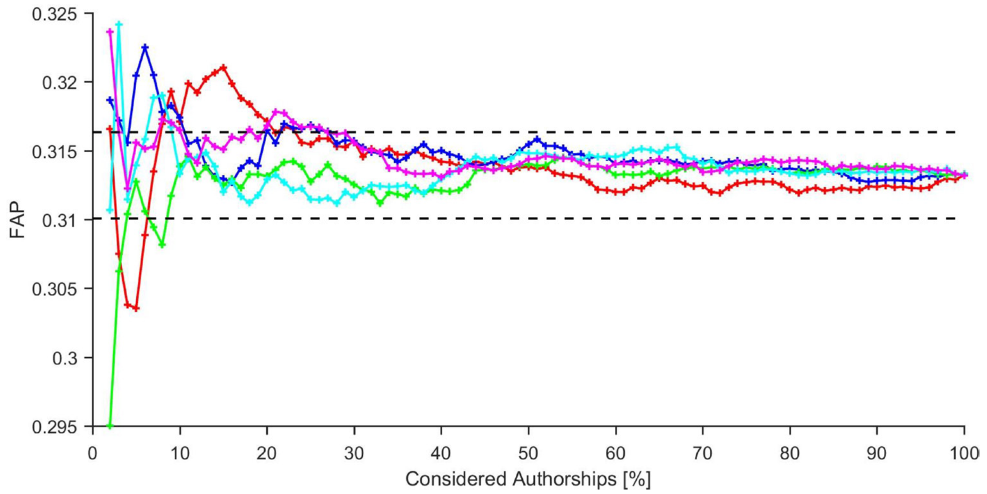

Supplementary Figure 6: Convergence of gender determination. The graph shows the determination of the FAP as a function of the considered authorships in 5 consecutive runs (authorships were shuffled before each run). The dashed lines represent the 1% interval around the effective FAP. Evidently, the algorithm converges very fast; it is sufficient to consider 10–25% of all *male* and *female* authorships to assess the FAP with an error deviation of less than 1%.

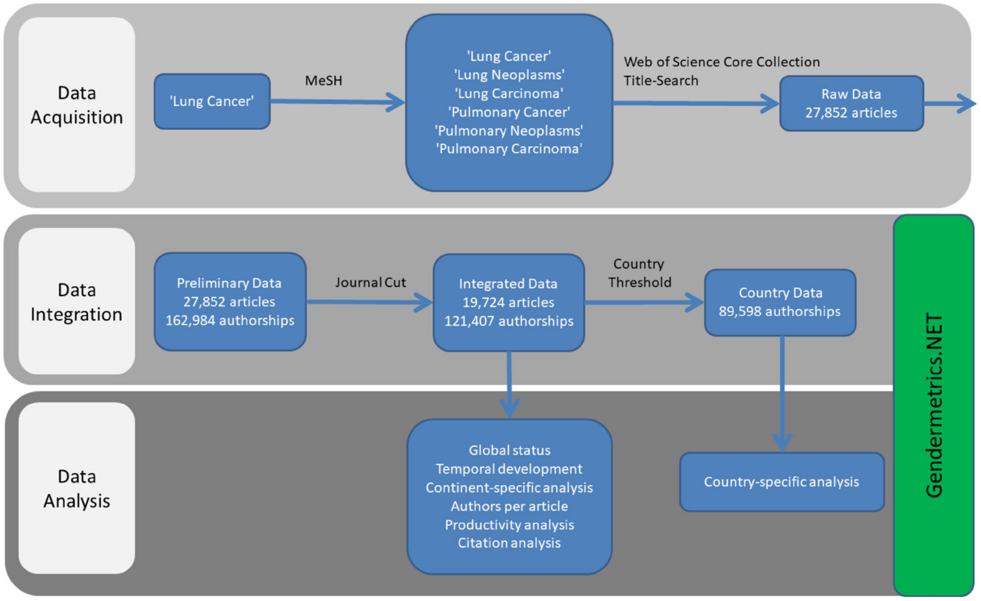

Supplementary Figure 7: Flow diagram of procedure steps. The synonyms for 'Lung Cancer' were determined by the MeSH library (Medical Subject Headings) of the National Library of Medicine. English-language research articles were acquired from the Web of Science Core Collection by performing a title search, yielding 27,852 articles. Data Integration and Data Analysis was conducted using Gendermetrics.NET [1]. Journals with a detection rate below 50% male or female authors were excluded from the analysis ('Journal Cut', in total 8128 articles). In order to ensure the validity of the country-specific analysis a gender detection threshold criterion for the inclusion of a country was applied. Specifically, countries with a detection fraction below 73.8% male and female authorships were excluded from this subanalysis.

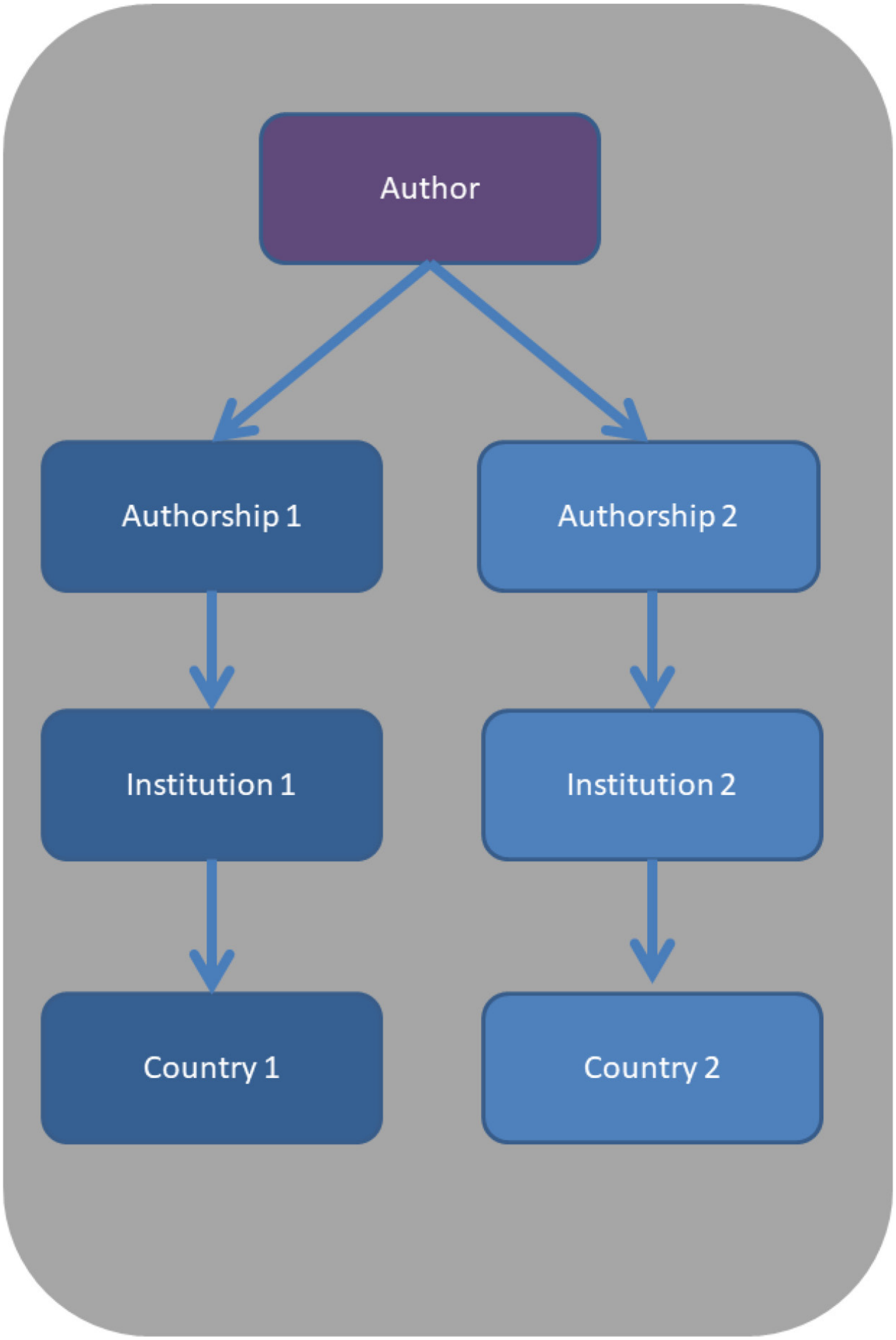

Supplementary Figure 8: The concept of authors and authorships. The research output of a country was determined on the basis of the associated institutions and their authorships. A single author is thus able to contribute to the research output of different countries.
